# Protein targeting to Starch 2 and the plastidial phosphorylase 1 revealed protein–protein interactions with photosynthesis proteins in yeast two-hybrid screenings

**DOI:** 10.1080/15592324.2025.2470775

**Published:** 2025-02-26

**Authors:** Sidratul Nur Muntaha, Joerg Fettke

**Affiliations:** Biopolymer Analytics, Institute of Biochemistry and Biology, University of Potsdam, Potsdam-Golm, Germany

**Keywords:** Starch, yeast two-hybrid, PTST2, PHS1, plastidial phosphorylase, photosynthesis, *Arabidopsis thaliana*

## Abstract

Starch metabolism in plants involves a complex network of interacting proteins that work together to ensure the efficient synthesis and degradation of starch. These interactions are crucial for regulating the balance between energy storage and release, adapting to the plant’s developmental stage and environmental conditions. Several studies have been performed to investigate protein–protein interactions (PPIs) in starch metabolism complexes, yet it remains impossible to unveil all of the PPIs in this highly regulated process. This study uses yeast-two-hybrid (Y2H) screening against the Arabidopsis leaf cDNA library to explore PPIs, focusing on the starch-granule-initiating protein named Protein Targeting to Starch 2 (PTST2, At1g27070) and the protein involved in starch and maltodextrin metabolism, namely, plastidial phosphorylase 1 (PHS1, EC 2.4.1.1). More than 100 positive interactions were sequenced, and we found chloroplastidial proteins to be putative interacting partners of PTST2 and PHS1. Among them, photosynthetic proteins were discovered. These novel interactions could reveal new roles of PTST2 and PHS1 in the connection between starch metabolism and photosynthesis. This dynamic interplay between starch metabolism and other chloroplast functions highlights the importance of starch as both an energy reservoir and a regulatory component in the broader context of plant physiology and adaptation.

## Introduction

Starch serves as the primary storage carbohydrate in the leaves, seeds, and storage organs of plants and is synthesized in plastids as insoluble, semicrystalline granules.^1–4^ While the molecular basis for starch polymer biosynthesis is well-studied and generally consistent between leaf and storage starches,^2^ we are only beginning to understand the mechanism of starch granule initiation in the chloroplast and the distinct roles of imperative or submissive protein–protein interactions (PPI) involved.

Studies suggest that starch-elongating enzymes, such as starch synthases (SSs, EC 2.4.1.2), are not able to utilize glucose or ADPG as the starting point for the generation of the glucan chain; instead, a primer-like initiation complex is used^5,6^ Furthermore, starch initiation appears to occur at specific plastid locations and is regulated by particular proteins.^7^ The elongation of soluble maltodextrins is a key step in this process, with chloroplastidial maltodextrin pools being utilized. It has been postulated that more than one starch initiation pathway exists and that one is dominant.^5,8^ In the dominant pathway, starch synthase 4 (SS4, At4g18240) and Protein Targeting to Starch 2 (PTST2, At1g27070.1) form the granule initiation complex. This complex is further processed by other starch synthases (SSs), branching enzymes (BEs, EC 2.4.18), and debranching enzymes (DBEs, EC 3.2.1.68) to ultimately generate a starch granule^5,6,9,10^ Starch synthase 4 (SS4, At4g18240) is identified as central to starch granule initiation. An immunoprecipitation study showed that PTST2 interacts with SS4 and influences granule initiation and morphology.^11^ There is evidence that PTST2 and its homologous protein PTST3 (AT5G03420)^11^ are critical during granule initiation, as they bind and deliver suitable maltodextrin primers to SS4 by conceding the helical structures via its coil domains and a carbohydrate-binding module (CBM).^11^ Recently, immunoprecipitation and mass spectrometry studies have revealed that PTST2 also interacts with two novel large coiled-coil domains containing plastidial proteins: MRC/PII1 (Myosin resembling chloroplast protein or protein involved in starch initiation 1; At4g32190) and MFP1 (MAR-binding filament-like protein 1; At3g1600.1).^11–13^ More recent exploration has revealed that starch initiation is triggered by MFP1, which interacts with PTST2 and plays a crucial role in determining the precise subchloroplastidial location of the starch granule initiation machinery.^7^ Additionally, MRC/PII1 is identified as a putative interacting partner of SS4 by using yeast-two-hybrid (Y2H) screening.^13^ Further studies revealed that MRC also binds to the non-catalytic starch synthase SS5 (At5g65685), suggesting that some of these interactions could be multivalent or mutually exclusive.^14^

Plastidial α-glucan phosphorylase 1 (PHS1 in Arabidopsis or Pho1 in other species) is the major active form of phosphorylase in plants, and, due to its reversible catalytic activity, it functions in both synthesizing and degradative pathways; however, its role varies across different species.^15^ It has been suggested that PHS1 may play a critical role in generating soluble maltodextrins during granule initiation, and it may provide substrates for the SS4 enzyme.^11,12^ In addition, evidence indicates that the plastidial phosphorylase interacts with branching enzymes (BEs) and disproportionating enzyme 1 (DPE1) to produce longer soluble maltodextrins^16,17^ with potential further interaction with SS4 to facilitate glucan chain elongation.^5^ Recent studies have shown that plastidial phosphorylase deficiency in potato and rice results in strong alterations in maltodextrin levels.^17,19^ which is required for the maintenance of normal granule phenotypes in potato.^20^ Furthermore, in collaboration with B-GRANULE CONTENT1 (BGC1), the plastidial phosphorylase specifically facilitates the initiation of small and round B-type granules in the wheat endosperm and is not essential for the initiation of big and flattened A-type granules.^21^ Moreover, analyzing the growth and metabolic profiles of the constitutive double mutants in Arabidopsis (in *phs1/mex1* and *phs1/dpe2*) provided insights into the potential role of the plastidial phosphorylase in transitory starch metabolism.^22,23^

Notably, a distinctive structural feature of the higher plant plastidial phosphorylase is the presence of an extra L80 region, which is a 50–82-amino-acid sequence long region of unknown function located near the middle of the primary sequences in various plant species.^24^ Findings indicate that it plays a role in modulating photosynthesis and starch metabolism, allowing for the fine-tuning of source and sink processes.^25^ However, the molecular mechanism of how the plastidial phosphorylase and its L80 region influence both photosystem 1 activity and starch metabolism remains unclear. Nevertheless, the involvement of the plastidial phosphorylase in the starch initiation, including its interactions with other proteins during this process, further complicates the existing protein–protein interaction (PPI) network, suggesting the potential involvement of additional proteins that are yet to be identified.

The aim of this study was to explore the PTST2 and PHS1 interacting partners through systemic yeast-two-hybrid (Y2H) screening.

## Materials and method

The cDNAs encoding AtPTST2 and AtPHS1, lacking their transit peptide, were cloned into the GAL plasmid pGBT9 and pGAD424, respectively (primer list S-1, Tables 1, 2).

**Table 1.** Interaction partners of AtPTST2 and AtPHS1.

AtPTS2 interaction partner					
Accession	Protein	Location	Total Score/bits	E-Value	Per-identity
AT3G16000.1	MFP1	Chloroplast	495	2e-171	99.64%
AT5G03420.1	PTST3	Chloroplast	330	9e-108	98.77%
AT3G54890.4	LHCA1	Chloroplast	312	8e-106	100%
AT5G05990	Mitocondrial glycoprotein family protein	Mitocondrial matrix, mitocondrion	410	2e-140	99.12%
AtPHS1 interaction partner					
AT1G09310	SVB-LIKE, SVB2, SVBL	Cytosol Nucleus	244	1e-80	100%
AT2G34420**	LHCB1.5, LHB1B2	Chloroplast	506	0.0	100%
AT1G29930**	LHCB1.3, AB140, CAB140, CAB1	Chloroplast	494	5e-178	97%
AT1G67870	Glycerin-rich protein	Extracellular region	257	5e-53	99.19%
AT2G47590	PHOTOLYASE/BLUE-LIGHT RECEPTOR 2, PHR2	nucleus	542	0.0	100%
AT1G29920**	AB165, CAB2, CHLOROPHYLL A/B-BINDING PROTEIN 2, LHCB1.1	Apoplast, Chloroplast, Chloroplast thylakoid membrane, thylakoid	512	0.0	100%
AT1G29910**	CAB3, LHCB1.2, AB180	Chloroplast	518	0.0	100%
AT2G34430**	DEG11, LHB1B1, LHCB1.4,	Chloroplast	508	0.0	100%
AT4G25640	ATDTX35, DTX35, FFT,	Chloroplast, cytoplasm	558	0.0	97.44
AT5G54270	LHCB3, LHCB3*1	Chloroplast	542	0.0	100%
At5g03340	ATPase, AAA-type, CDC48 Protein;	Golgi apparatus, plant-type cell wall, plasma membrane	643	0.0	99.69%
AT4G24770	ATRBP31, ATRBP33, CP31, CP31A, RBP31	Chloroplast, cytosol	164	1e-64	100%

Protein identification method was NCBI BLAST and TAIR BLAST. Per identity and total score in NCBI BLAST refer to the percentage of identical base pairs or amino acids between two sequences in an alignment; NCBI BLAST total score and TAIR BLAST bit score refer the some of the score from all aligned segments between the query and the subject sequence and a normalized score representing the quality of a single segment, respectively. Lower E-values (<1e-5) and higher bit score (>200) indicate a higher probability of true interactions.^26,27^

**Homologous proteins.

The yeast-two-hybrid (Y2H) technique was based on the ‘*Matchmaker*^*TM*^ *GAL4 Two – Hybrid System 3 & Libraries User Manual*’ and the ‘*Yeast Protocols Handbook*’ (both Clontech, Heidelberg, Germany). Experiments were set up for separate screenings of AtPTST2 and AtPHS1 against Arabidopsisis cDNA library, as well as direct interaction between AtPTST2 and AtPHS1 fragments. The bait vectors were fused to the GAL4 DNA-binding domain (BD) encoded by the vector pGBT9 following polyethylene glycol/lithium acetate (PEG/LiAc)-mediated transformation system in yeast strain Y187,^28–31^ and the prey vector pAD-Gal4 – cDNA library in Y190 strain was prepared and used according to the following guideline: www.agilent.com/cs/library/usermanuals/public/235611.pdf.

### Yeast transformation method

In a glass tube, 8 mL of YPAD medium was inoculated with 2 μL yeast cells (yeast strain Y187). The tube was incubated overnight at 30°C with shaking at 220 rpm, and the following day, bait plasmids were transformed into the yeast cell. In a small conical flask, 50 mL of YPAD medium with 2–4 mL of the overnight culture was inoculated and then incubated for 3–4 h at 30°C with shaking (220 rpm). Afterward, the culture was placed in a 50 mL falcon tube and centrifuged for 5 min at 3500 rpm. The supernatant was discarded, and the pellet was washed in 25 mL of deionized water. The previous centrifugation step was repeated. The resulting pellet was gently dissolved in 1 mL 100 mm of lithium acetate (LiAc) and transferred into a 1.5 mL tube. After a short centrifugation of the tube at 13,000 rpm, the supernatant was discarded, and the pellet was dissolved in approximately 100 μL of 100 mm LiAc via gentle pipetting. The final volume of about 400–500 μL was split into several tubes; ultimately, each tube contained 50 μL of the suspension. After the short centrifugation of the tubes, the supernatant was discarded, and reaction mixture (50% [w/v] PEG − 240 μL, 1 M LiAc – 36 μL, Salmon sperm carrier DNA − 5 μL, Bait plasmid pGBT9-PTST2 or pGBT9-PHS1 – ~25 ng 2.5 μL, water – 74 μL) was added to each tube. Each of these transformation mixtures was vortexed for 1 min and incubated for 30 min at 30°C before the cells were heat shocked for 20 min at 42°C. After quickly centrifuging the tubes at 13,000 rpm, the supernatant was discarded, and the pellet was dissolved in 400 μL of water via pipetting. About 200 μL of cell suspension was plated on a SCAD-Trp plate, and the plates were incubated for 2–3 days at 30°C, allowing the yeast cells to grow.

### Two-hybrid mating

In a tube, 4 mL of SCAD-trp with cells from yeast bait plasmid plates was inoculated and then incubated overnight at 30°C. The next day, 150 mL of SCAD-trp with 1–2 mL of overnight culture was inoculated and then incubated overnight at 30°C. The following day, the culture was transferred in a falcon tube following centrifugation for 5 min at 4,000 rpm. After discarding the supernatant, the pellet was washed in 10 mL water, and the previous centrifugation step was repeated. The library cell (pAD10-cDNA library in Y190, used the protocol form www.agilent.com/cs/library/usermanuals/public/235611.pdf.) was thawed and resuspended in 20 mL of YPAD medium following incubation at 30°C. Afterward, the pellet of the yeast bait culture and the library cell suspension were combined. The combination mixture was vortexed before centrifuging for 5 min at 4,000rpm. After discarding the supernatant, the pellet was resuspended in 2 mL of residual liquid. About 400–500 µL of aliquots were platted in five plates of YPAD medium and incubated for 4–5 h at 30°C. Afterward, the cells from the YPAD plates were washed with 2 × 3 mL YPAD liquid medium, and the suspension was combined. Following the centrifugation step, the pellet was collected and washed in 15 mL water; then, the centrifugation step was repeated, the supernatant was discarded, and the pellet was resuspended in 5.3 mL of water. This was then plated out in large SCAD-LTH 4 mm 3-AT plates (~1 mL aliquot in each plate) and incubated for 5–7 days at 30°C. A dilution series with the rest of the cell culture (10^−2^, 10^−3^) was prepared, and 100 µL of the dilutions were placed on small SCAD-LT plates and incubated for 2 days at 30°C to assess the mating efficiency (S-2). After about 1 week, when single colonies were grown in large SCAD-LTH-4 mm 3-AT plates, positive colonies were picked and streaked on small SCAD-LTH-25 mm 3-AT plates, SCAD-LTH-4 mm 3-AT plates, and SCAD-LTH-4 mm 3-AT plates with nitrocellulose membrane. All the plates were incubated for 2–3 days at 30°C. The *lacZ* activity was tested using the nitrocellulose membrane. To prepare 5X *LacZ* buffer (100 mL), 5.34 g Na₂HPO₄ (300 mM), 2.76 g NaH₂PO₄ (200 mM), 0.375 g KCl (50 mM), and 0.125 g MgSO₄ (5 mM) were combined, and pH was adjusted to 7.0 with NaOH. For *LacZ* assay buffer (5 mL), 1 mL of 5X LacZ buffer, 167 μL of 2% [w/v] X-gal in DMF, and 13.5 μL of β-mercaptoethanol (0.27% [v/v]) were mixed.

Per tested membrane, two Whatman^TM^ Chromatography Papers (GE Healthcare, Freiburg, Germany) the same size of the Petri dish used were placed into the dish, and the buffer was added until the Whatman paper was completely wet. In parallel, the nitrocellulose membrane with the yeast colonies was frozen for at least 10 s in liquid nitrogen and then transferred to a room temperature environment for about 1 min to permeabilize the cells. Afterward, the membrane was placed in the Petri dish and incubated at 30°C. The colonies started to turn blue after about 20 min. The plates were incubated for up to 1 h before the result was documented.

### Yeast plasmid extraction

To extract yeast plasmid from SCAD-LTH-25 mm 3-AT plates, each single, positive colony was picked and inoculated in 2 mL of SCAD-LT liquid medium and incubated overnight at 30°C. The next day, the culture was transferred to a 2 mL tube and centrifuged for 1 min at 13,000 rpm. After discarding the supernatant, the pellet was resuspended in 200 µL of lysis buffer, and then 200 µL of glass beads (0.4–0.6 mm) and 200 µL of phenol-chloroform (1:1, v/v) were added. The mixture was vortexed and centrifuged for 10 min at 13,000 rpm to separate pellet from the cloudy suspension in the upper layer. About 200 µL of suspension was transferred to a new tube, and 500 µL of cold ethanol and 1/10 Vol (~20 µL) 5 M LiCI were added. Then, the tube was frozen thoroughly in liquid nitrogen. Afterward, the pellet was collected by centrifuging for 10 min at 13,000 rpm. The pellet was washed in 250 µL of 70% [v/v] ethanol and then centrifuged for 5 min, and the supernatant was discarded. The pellet was dried for 20 min at 37°C and then dissolved in 20–30 µL of H_2_O.

### Transformation of yeast plasmid in *E. coli* acterial cell and selection of Leu- colonies

Each yeast plasmid was transformed into electro-competent KC8 cells by electroporation and then plated out on LB-Amp plate and incubated overnight at 37°C to grow single colonies. Each positive colony was picked and streaked on M9 selection medium (M9-leu and M9-trp) and incubated at 37°C for 1–2 days. The colony that was grown on both plates (M9-leu and M9-trp); it was a positive colony and was isolated for extraction of the plasmid DNA.

### Analysis of isolated AD:cDNA plasmids

Each positive colony was picked from M9-leu plate, inoculated in 2 mL of LB-Amp medium, and incubated overnight at 37°C. The following day, plasmid was extracted in accordance with.^32^ Afterward, each plasmid was separately transferred to *E. coli* DH5α chemically competent cell, and plasmids were extracted by Thermo Fischer miniprep kit. Test digestion and agarose gel electrophoresis were performed for each plasmid. Then, each plasmid was sequenced by LGC genomics (https://shop.lgcgenomics.com/.) using the primer 5’ -TAC CAC TAC AAT GGA TG −3’ for pGAD10 vector. Finally, the obtained sequences were analyzed by the NCBI BLAST 2.9.0+ algorithm (https://blast.ncbi.nlm.nih.gov.) and **TAIR BLAST 2.9.0**+.

### Direct interaction assay

Direct interaction was performed according to the pevious study.^33^ The interaction between PTST2 and PHS1 was tested by co-transforming the respective plasmid into yeast strain Y190 (Experimental setup, S-1, Table 4, 5). Transformants were selected from the Leu- and Trp-deficient medium at 30°C for 3 days, then transferred to His-, Leu-, and Trp-deficient medium with 25 mm 3-AT for growth selection. *LacZ* activity was performed to confirm protein interaction.

## Result

### Protein–protein interactions of AtPTST2 and AtPHS1

We utilized a sequential transformation system. Baits were fused to the GAL4 DNA binding domain (BD), and as prey, we used the cDNA library fused to pAD GAL4.^18^ After mating (Experimental setup, S-1, Tables 3), the cells were placed in a SCAD-LTH 4 mm 3-AT Plate and incubated at 30°C. We began monitoring the transformation plates for colony growth after 4 days of incubation, and positive colonies were selected for up to 3 weeks.

**Figure 1.**
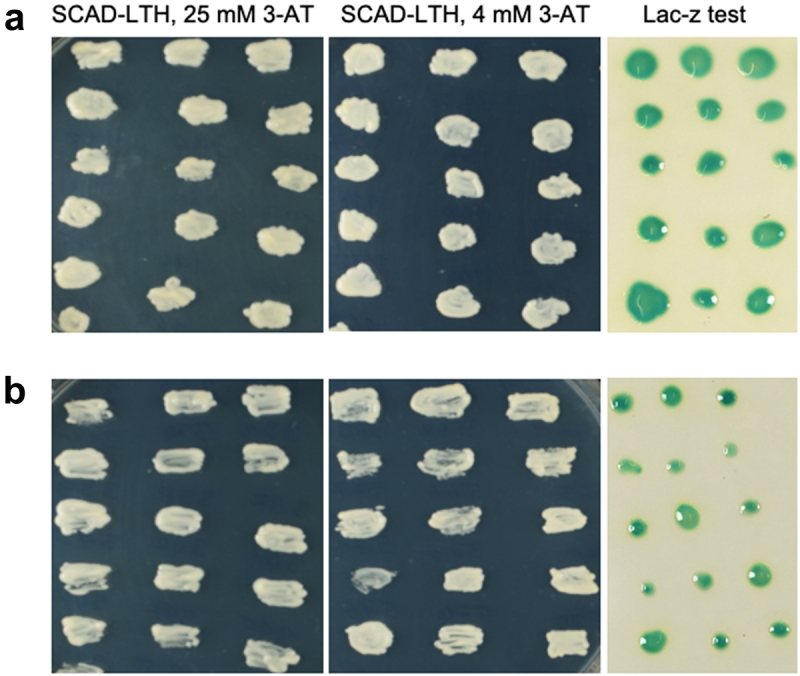
Verification of putative positive clones. Picking and streaking the Y2H positives from SCAD-LTH 4 mm 3-AT transformation plate to replica plates and verifying positive clone via *LacZ* assay (15 positive clones are shown as representative plates for each bait). (a) AtPTST2 and (b) AtPHS1.

Positive colonies were selected meticulously. Each colony’s growth was observed over several days, and large ones that actively grew on plates were transferred to the selective medium (SCAD-LTH +25 mm 3-AT, SCAD-LTH +4 mm 3-AT, SCAD-LTH +25 mm 3-AT with NC membrane); meanwhile, small colonies were generally avoided. Overall, about 160 positive clones (110 colonies from the AtPTST2 –cDNA library and 50 from the AtPHS1 – cDNA library) were picked and streaked on SCAD-LTH +3-AT plates to confirm reporter gene activation (Figure 1). After performing a positive *LacZ* assay for each clone, the plasmid was extracted separately, transformed into individual bacterial cells (Kc8 strain), and plated into the LB-Amp medium. After 16 h of incubation, the clones were grown on selective media M9-Leu and M-Trp, and all positive clones were checked via restriction analyses following sequencing and sequence alignment.

For PTST2, an interaction with PTST3 and MFP1 was detected, confirming already published results.^11,12^ In addition, we found an interaction between PTST2 with the Light Harvesting Complex 1 protein (LHCA1) (Table 1).

In the case of PHS1, no interaction with known starch metabolism proteins was detected. However, we detected an interaction with previously known chloroplast proteins as new interaction partners. Notably, six of them belonged to the Light Harvesting Complex family, which is part of a larger super complex of photosystems (Table 1). The corresponding cDNA sequences are given in S-3, Table 6. For AtPHS1, we found five proteins that are homologous (S-4, Table 7).

Other interactions were physiologically insignificant as the location of the interacting protein was outside the chloroplast; thus, other isoforms or homologous proteins may be of interest here. A direct interaction between PTST2 and PHS1 was not observed, not even in a direct interaction assay (data not shown).

## Discussion

Currently, we rely on distinct locations and functions of PTST2 and PHS1 and photosynthetic proteins. However, a recent study revealed that the Arabidopsis plastid-nucleus-located protein WHIRLY1 interacts with the photosynthetic protein LHCA1, altering the photochemical activities of photosystem I (PSI) and eliciting light adaptation in Arabidopsis.^34^ More recent studies have identified that Pho1 in rice interacts with PsaC, a subunit of photosystem 1, indicating an additional role of Pho1 beyond starch biosynthesis by modulating photosynthetic activity^24,25^ It could be hypothesized that the activities of PTST2, PHS1, and photosynthetic proteins may be coordinated to ensure efficient energy utilization and the storage of starch in plants.

### Physical proximity and compartmentalization between thylakoid membrane and stroma

Although LHCA1 is embedded in the thylakoid membrane and starch metabolism occurs in the stroma, there could be physical proximity in certain specialized regions of the chloroplast where membrane-bound processes interface with soluble metabolic pathways.

### Protein complexes and membrane contact sites

Starch-related proteins PTST2 and PHS1 might be parts of larger, dynamic protein complexes that transiently interact with thylakoid membrane components. For instance, PTST2 in Arabidopsis has been identified as a dual localized protein partially associated with the thylakoid membranes in wild-type plants. Interestingly, its localization changes in the absence of MFP1, with the protein then residing completely in the chloroplast stroma.^7^ Additionally, rice Pho1 interacts with PsaC, a subunit of photosystem I (PSI).^25^ A recent study suggests the presence of membrane contact sites (MCSs) in different chloroplast compartments,^35^ which could facilitate direct interactions between membrane-bound (photosynthetic proteins) and soluble proteins (PTST2 and PHS1).

### Regulatory Mechanisms

Post-translation modifications, such as phosphorylation, may regulate the interactions between photosynthetic proteins and starch metabolism proteins. Such interactions can act as switches, facilitating or inhibiting PPIs in response to environmental or metabolic cues. However, this is probably not (fully/correctly) established in the yeast system, resulting in the incomplete detection of possible PPIs. One possible example of this is the case of PTST2 and PHS1. No evidence was found for direct interaction with PTST2 and PHS1, albeit the double mutant *phs1ptst2* revealed altered starch metabolism.^36^

Interestingly, it has been demonstrated that there is a coordination of plastid and light-signaling pathways during Arabidopsis leaves development under different photoperiods.^37^

### Energy and Metabolite Exchange

The energy produced in photosystems is utilized in the stroma for ATP and NADPH production, which are essential for starch biosynthesis. Metabolic crosstalk might involve transient interactions or signaling molecules linking these processes.^38^

Overall, we herein present further evidence for the interaction/coordination of the primary plant metabolism, i.e., starch metabolism-related processes and the initial energy production – photosynthesis, via already known proteins.

## Supplementary Material

suppl_data_corrected.docx

## References

[1] Compart J, Li X, Fettke J. Starch-A complex and undeciphered biopolymer. J Plant Physiol. 2021;258-259:153389. doi:10.1016/j.jplph.2021.153389. 33652172

[2] Smith AM, Zeeman SC. Starch: a flexible, adaptable carbon store coupled to plant growth. Annu Rev Plant Biol. 2020;71(1):217–6. doi:10.1146/annurev-arplant-050718-100241. 32075407

[3] Seung D. Amylose in starch: towards an understanding of biosynthesis, structure and function. New Phytol. 2020;228(3):1490–1504. doi:10.1111/nph.16858. 32767769

[4] Bertoft E. Understanding starch structure: recent progress. Agronomy. 2017;7(3):56. doi:10.3390/agronomy7030056.

[5] Malinova I, Qasim HM, Brust H, Fettke J. Parameters of starch granule genesis in chloroplasts of Arabidopsis thaliana. Front Plant Sci. 2018;9:761. doi:10.3389/fpls.2018.00761. 29922326 PMC5996153

[6] Mérida A, Fettke J. Starch granule initiation in Arabidopsis thaliana chloroplasts. Plant J Cell Mol Biol. 2021;107(3):688–697. doi:10.1111/tpj.15359. 34051021

[7] Sharma M, Abt MR, Eicke S, Ilse TE, Liu C, Lucas MS, Pfister B, Zeeman SC. MFP1 defines the subchloroplast location of starch granule initiation. Proc Natl Acad Sci USA. 2024;121(3):e2309666121. doi:10.1073/pnas.2309666121. 38190535 PMC10801857

[8] Malinova I, Alseekh S, Feil R, Fernie AR, Baumann O, Schöttler MA, Lunn JE, Fettke J. Starch synthase 4 and plastidal phosphorylase differentially affect starch granule number and morphology. Plant Physiol. 2017;174(1):73–85. doi:10.1104/pp.16.01859. 28275148 PMC5411139

[9] Nakamura Y. Ed. Starch: metabolism and structure. Springer Tokyo: Springer; 2015 doi:10.1007/978-4-431-55495-0.

[10] Seung D, Smith AM. Starch granule initiation and morphogenesis—progress in Arabidopsis and cereals. J Exp Botany. 2019;70(3):771–784. doi:10.1093/jxb/ery412. 30452691

[11] Seung D, Boudet J, Monroe J, Schreier TB, David LC, Abt M, Lu KJ, Zanella M, Zeeman SC. Homologs of protein targeting to starch control starch granule initiation in Arabidopsis leaves. The Plant Cell. 2017;29(7):1657–1677. doi:10.1105/tpc.17.00222. 28684429 PMC5559754

[12] Seung D, Schreier TB, Bürgy L, Eicke S, Zeeman SC. Two plastidial coiled-coil proteins are essential for normal starch granule initiation in arabidopsis. The Plant Cell. 2018;30(7):1523–1542. doi:10.1105/tpc.18.00219. 29866647 PMC6096604

[13] Vandromme C, Spriet C, Dauvillée D, Courseaux A, Putaux JL, Wychowski A, Krzewinski F, Facon M, D’Hulst C, Wattebled F. PII1: a protein involved in starch initiation that determines granule number and size in Arabidopsis chloroplast. The New Phytol. 2019;221(1):356–370. doi:10.1111/nph.15356. 30055112

[14] Abt MR, Pfister B, Sharma M, Eicke S, Bürgy L, Neale I, Seung D, Zeeman SC. STARCH SYNTHASE5, a noncanonical starch synthase-like protein, promotes starch granule initiation in arabidopsis. Plant Cell. 2020;32(8):2543–2565. doi:10.1105/tpc.19.00946. 32471861 PMC7401018

[15] Shoaib N, Liu L, Ali A, Mughal N, Yu G, Huang Y. Molecular functions and pathways of plastidial starch phosphorylase (PHO1) in starch metabolism: current and future perspectives. Int J Mol Sci. 2021;22(19):10450. doi:10.3390/ijms221910450. 34638789 PMC8509025

[16] Nakamura Y, Ono M, Sawada T, Crofts N, Fujita N, Steup M. Characterization of the functional interactions of plastidial starch phosphorylase and starch branching enzymes from rice endosperm during reserve starch biosynthesis. Plant Sci: Int J Exp Plant Biol. 2017;264:83–95. doi:10.1016/j.plantsci.2017.09.002. 28969805

[17] Dong X, Chen L, Yang H, Tian L, Dong F, Chai Y, Qu LQ. Pho1 cooperates with DPE1 to control short maltooligosaccharide mobilization during starch synthesis initiation in rice endosperm. TAG Theor Appl Genet Theoretische und Angew Genetik. 2023;136(3):47. doi:10.1007/s00122-023-04250-z. 36912930

[18] Fields S, Song O. A novel genetic system to detect protein-protein interactions. Nature. 1989;340(6230):245–246. doi:10.1038/340245a0. 2547163

[19] Flores-Castellanos J, Fettke J. The plastidial glucan phosphorylase affects the maltooligosaccharide metabolism in parenchyma cells of potato (Solanum tuberosum L.) tuber discs. Plant Cell Physiol. 2023;64(4):422–432. doi:10.1093/pcp/pcac174. 36542813 PMC10109208

[20] Sharma S, Friberg M, Vogel P, Turesson H, Olsson N, Andersson M, Hofvander P. Pho1a (plastid starch phosphorylase) is duplicated and essential for normal starch granule phenotype in tubers of Solanum tuberosum L. Front Plant Sci. 2023;14:1220973. doi:10.3389/fpls.2023.1220973. 37636090 PMC10450146

[21] Kamble NU, Makhamadjonov F, Fahy B, Martins C, Saalbach G, Seung D. Initiation of B-type starch granules in wheat endosperm requires the plastidial α-glucan phosphorylase PHS1. Plant Cell. 2023;35(11):4091–4110. doi:10.1093/plcell/koad217. 37595145 PMC10615211

[22] Malinova I, Mahlow S, Alseekh S, Orawetz T, Fernie AR, Baumann O, Steup M, Fettke J. Double knockout mutants of Arabidopsis grown under normal conditions reveal that the plastidial phosphorylase isozyme participates in transitory starch metabolism. Plant Physiol. 2014;164(2):907–921. doi:10.1104/pp.113.227843. 24302650 PMC3912115

[23] Li X, Apriyanto A, Flores Castellanos J, Compart J, Muntaha SN, Fettke J. Dpe2/phs1 revealed unique starch metabolism with three distinct phases characterized by different starch granule numbers per chloroplast, allowing insights into the control mechanism of granule number regulation by gene co-regulation and metabolic profiling. Front Plant Sci. 2022;13, Article 1039534. 10.3389/fpls.2022.1039534. PMC9667719 36407636

[24] Hwang SK, Koper K, Okita TW. The plastid phosphorylase as a multiple-role player in plant metabolism. Plant Sci: Int J Exp Plant Biol. 2020;290:110303. doi:10.1016/j.plantsci.2019.110303. 31779913

[25] Koper K, Hwang SK, Wood M, Singh S, Cousins A, Kirchhoff H, Okita TW. The rice plastidial phosphorylase participates directly in both sink and source processes. Plant & Cell Physiol. 2021;62(1):125–142. doi:10.1093/pcp/pcaa146. 33237266

[26] Altschul SF, Madden TL, Schäffer AA, Zhang J, Zhang Z, Miller W, Lipman DJ. Gapped BLAST and PSI-BLAST: a new generation of protein database search programs. Nucleic Acids Res. 1997;25(17):3389–3402. doi:10.1093/nar/25.17.3389. 9254694 PMC146917

[27] Pearson WR. An introduction to sequence similarity (“homology”) searching. Curr Protocol In Bioinf. 2013;42(1):.3.1.1–.3.1.8. doi:10.1002/0471250953.bi0301s42. PMC3820096 23749753

[28] Ito H, Fukuda Y, Murata K, Kimura A. Transformation of intact yeast cells treated with alkali cations. J Bacteriol. 1983;153(1):163–168. doi:10.1128/jb.153.1.163-168.1983. 6336730 PMC217353

[29] Schiestl RH, Gietz RD. High efficiency transformation of intact yeast cells using single stranded nucleic acids as a carrier. Curr Genet. 1989;16(5–6):339–346. doi:10.1007/BF00340712. 2692852

[30] Gietz D, St Jean A, Woods RA, Schiestl RH. Improved method for high efficiency transformation of intact yeast cells. Nucleic Acids Res. 1992;20(6):1425. doi:10.1093/nar/20.6.1425. 1561104 PMC312198

[31] Kawai S, Hashimoto W, Murata K. Transformation of Saccharomyces cerevisiae and other fungi: methods and possible underlying mechanism. Bioengineered Bugs. 2010;1(6):395–403. doi:10.4161/bbug.1.6.13257. 21468206 PMC3056089

[32] Hummel S. Ancient DNA typing: methods, strategies, and applications. Springer-Verlag Berlin Heidelberg New York: Springer; 2003. doi:10.1007/978-3-662-05050-7.

[33] Nietzsche M, Schießl I, Börnke F. The complex becomes more complex: protein-protein interactions of SnRK1 with DUF581 family proteins provide a framework for cell- and stimulus type-specific SnRK1 signaling in plants. Front Plant Sci. 2014;5:54. doi:10.3389/fpls.2014.00054. 24600465 PMC3930858

[34] Huang D, Lin W, Deng B, Ren Y, Miao Y. Dual-located WHIRLY1 interacting with LHCA1 alters photochemical activities of photosystem I and Is involved in light adaptation in arabidopsis. Int J Mol Sci. 2017;18(11):2352. doi:10.3390/ijms18112352. 29112140 PMC5713321

[35] Wang D, He H, Wei C. Cellular and potential molecular mechanisms underlying transovarial transmission of the obligate symbiont sulcia in cicadas. Environ Microbiol. 2023;25(4):836–852. doi:10.1111/1462-2920.16310. 36515176

[36] Singh A, Rajendran R, Schöttler MA, Li X, Liu Q, Muntaha SN, Fettke J. The plastidial glucan phosphorylase modulates maltodextrin metabolism and affects starch parameters in Arabidopsis thaliana. J Exp Botany. 2025; eraf041. Advance online publication. 10.1093/jxb/eraf041. 39883045 PMC12116187

[37] Lepistö A, Rintamäki E. Coordination of plastid and light signaling pathways upon development of Arabidopsis leaves under various photoperiods. Mol Plant. 2012;5(4):799–816. doi:10.1093/mp/ssr106. 22199239 PMC3399700

[38] Geigenberger P, Fernie AR. Metabolic control of redox and redox control of metabolism in plants. Antioxid & Redox Signaling. 2014;21(9):1389–1421. doi:10.1089/ars.2014.6018. PMC4158967 24960279

