# Supplementary material for: Protein targeting to Starch 2 and the plastidial phosphorylase 1 revealed protein–protein interactions with photosynthesis proteins in yeast two-hybrid screenings
Source: Plant Signal Behav. 2025 Feb 26;20(1):2470775. doi: 10.1080/15592324.2025.2470775 (PMC11866963; doi:10.1080/15592324.2025.2470775)
Supplement: suppl_data_corrected.docx [file KPSB_A_2470775_SM3692.docx]

**S-1: Primer for PGBT9-PTST2 and PGBT9- PHS1 plasmid:**  The bold letters of the primer sequence indicate the recognition sequence of the restriction enzyme used for cloning.

Table 1: List of primers with restriction enzymes used for the fragments of AtPTST2.

| Forward primer with BamHI | | CG**GGATCC**GTTGTAATGGGGTTTCGAGGATG |
| --- | --- | --- |
| Reverse primer with PstI | AA**CTGCAG**TCAAGAGATGATTAGCAGATTG | |

Table 2: List of primers with restriction enzymes used for the fragments of AtPHS1.

| Forward primer with EcoRI | CGCTGC**GAATTC**AGCGAACCGAAAGCCAAAGT |
| --- | --- |
| Reverse primer with SmaI | CAGTTC**CCCGGG**TCATGGAAGTTCCACTTGCTTA |

Table 3. Experimental setup for library screening.

| Bait plasmid | | Prey Plasmid |
| --- | --- | --- |
| 2. | pGBT9-PTST2 | pAD10-cDNA library |
| 2 | pGBT9-PHS1 | pAD10-cDNA library |

Table 4. Experimental setup for direct interaction.

| Bait plasmid | | Prey Plasmid |
| --- | --- | --- |
| 1. | pGBT9-PHS1 | pGAD424-PTST2 |
| 2. | pGBT9-PTST2 | pGAD424-PHS1 |

Table 5. Experimental setup for control of direct interaction.

| 1. | pGBT9-PTST2 | pGAD424 empty |
| --- | --- | --- |
| 2. | pGBT9 empty | pGAD424-PTST2 |
| 3. | pGBT9-PHS1 | pGAD424 empty |
| 4. | pGBT9 empty | pGAD424-PHS1 |

**S-2: Efficiency test.** Control plates used to determine the number of colony-forming units to calculate the efficiency of transformation and library titer for the PHS1 screening. (A-B) 1000 (A)

and 10,000-fold (B) dilutions of the transformed yeasts were plated out on medium lacking

the Leu and Trp for selection for bait and prey plasmids, respectively.


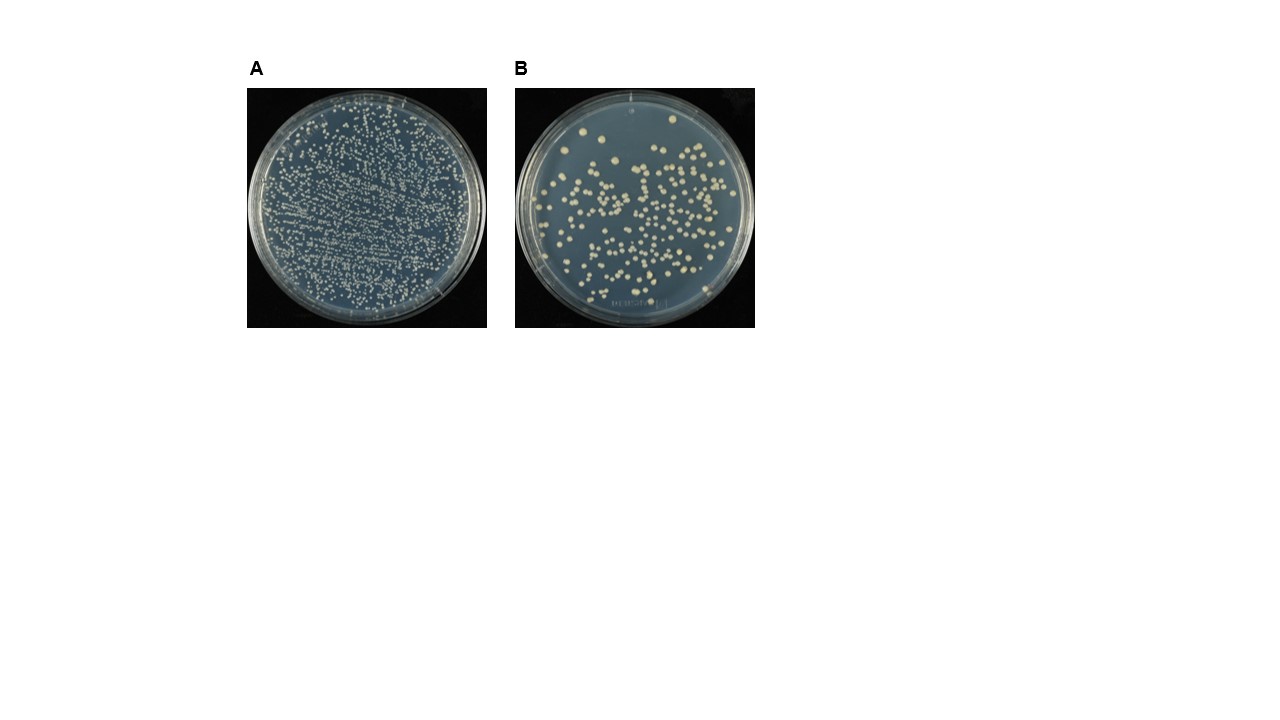


**S-3:**

Table 6. The corresponding cDNA sequences of identified proteins.

| **Bait protein** | **Identified protein’s Accession** | **cDNA sequence** |
| --- | --- | --- |
| **AtPTST2** | AT3G54890.4 | TACGTAGATTCTGCTTTTATCTGAACCACATAGTGCAGATGGATATCGAACAGAAGAACATGATATCGAGAAGACACGTGGCCCAATGAAAAAGCCACGCAGGCTCTGTATAGCCAATTGCAATCTGGTTTTAGATTCTCATCTCACAAATCTTACTTATACGATCAGTCTCCCACATTCACACTTCCACCAAACACCTCTCGTCCACATCTTTCTCCTTTGGTGGCAAACACAACGACAGATAGAGAGAAACGATGGCGTCGAACTCGCTTATGAGCTGTGGCATAGCCGCCGTGTACCCTTCGCTTCTCTCTTCTTCCAAGTCTAAATTCGTATCCGCCGGAGTTCCACTCCCAAACGCCGGGAATGTTGGTCGTATCAGAATGGCTGCTCACTGGATGCCTGGCGAGCCACGACCAGCTTACCTTGACGGTTCTGCTCCTGGTGACTTTGGGTTTGACCCACTTGGACTTGGAGAAGTTCCAGCGAACCTTGAGAGATACAAAGAGTCAGAGCTCATCCACTGTAGATGGGCTATGCTCGCTGTTCCTGGGATTTTGGTACCAGAAGCATTAGGATATGGAAACTGGGTTAAGGCTCAGGAATGGGCAGCACTACCAGGGGGTCAAGCCACTTACTTGGGAAACCCAGTCCCGTGGGGTACTTTGCCCACAATCTTGGCCATTGAGTTCTTAGCCATTGCATTTGTTGAGCACCAGAGAAGTATGGAGAAAGACCCTGAGAAGAAGAAGTACCCGGGAGGCGCATTTGACCCTCTTGGATACTCGAAGGACCCCAAGAAGCTCGAGGAATTGAAAGTTAAAGAGATCAAGAACGGGCGGCTTGCGCTGTTGGCGTTTGTAGGATTCTGTGTGCAACAGTCGGCTTACCCGGGGACAGGACCATTGGAGAACTTGGCAACTCACTTGGCGGATCCATGGCACAACAACATTGGCGATATTGTTATCCCTTTCAACTAATGAATGTAAAAATAGAAATATGTGTACCTTATGAGCTTTATGTGTATCAAAAACACTGTGTAATGAAAGACAGATTTGTGAATTCCTGTAATTTGTCTTGGTGATCGAATGCTCGTGTGAGCTTGAATTCTAAGTGATGAACCTTTTGAACCCAAATACACATTACATCTCATGCTTCCTACAGAAGAAACATTGTAAGCTC |
| **AtPHS1** | AT4G24770 | ATAGACTTTGTGTATTATTTTTTTATAATATAAACTTCGTTTTGATAAGTTTATATAAACCTTGTTTTCGTAAGAGATTGTGAAATTTTCTTTGCAGAGGGACATGGTTATTTTTTGTCTTTGGTTCTTGTAATGAGATTTTAACTATGGTAAATCTAAGAGCCCATTTGAAGATGATGTCTCGGCCCTCCTTATCCTTATCTCGCCCACCGTTCTAAAACCCTTCCCTTCCTCTTCTTTTCTCTTCTCCTTCCACTTCCACTTCTTCTATCTCTCACAATTCAGCAATGGCTTCTTCTATAGTTACCTCTAGCTTGAAGCCTTTAGCCATGGCCGATTCTTCCTCTTCTACCATTTTCTCCCATCCTTCCATCTCCTCTACCATCTCTTCCTCCAGAATTCGCAGCTCCAGTGTTTCCCTCCTTACCGGACGCATTAACCTGCCCCTCTCTTTCTCTCGCGTCTCTCTATCTCTTAAAACCAAAACCCACCTTAAAAAATCCCCCTTTGTCTCCTTCGTTGCCCAGACTTCGGATTGGGCTGAAGAAGGTGGAGAAGGAAGCGTCGCTGTTGAGGAGACCGAGAACAGTTTAGAGTCACAAGATGTGAGCGAAGGAGATGAGAGCGAAGGAGATGCGAGCGAAGGAGATGTGAGCGAAGGAGATGAGAGCGAAGGAGATGTGAGCGAAGGAGCTGTGAGCGAAAGAGCTGAGTTCCCGGAGCCATCGGAAGAAGCCAAGCTTTTCGTCGGAAATTTGGCTTATGATGTTAATAGCCAAGCTTTGGCTATGCTCTTTGAGCAAGCCGGTACTGTTGAAATCGCCGAGGTTATATACAATAGGGAAACTGACCAGAGTCGTGGGTTTGGATTTGTGACAATGAGTTCTGTGGACGAAGCTGAGACAGCTGTGGAGAAGTTCAACCGTTATGATCTTAACGGACGTCTACTAACTGTAAACAAGGCAGCTCCAAGAGGATCACGTCCAGAACGTGCGCCTCGGGTATATGAACCTGCATTCAGAGTATATGTAGGGAATCTACCATGGGATGTGGACAATGGCCGTCTAGAACAGCTTTTCAGTGAGCATGGTAAAGTTGTGGAAGCTAGGGTGGTTTACGACCGTGAGACAGGTCGTTCACGTGGATTCGGGTTTGTCACAATGTCTGACGTGGATGAACTCAACGAAGCCATCTCTGCCCTCGATGGACAGAACTTGGAGGGTAGAGCAATCAGAGTGAATGTAGCGGAAGAGCGTCCTCCAAGGCGTGGATATTAAAGCTGAGATCTAGCTGTGCCCTCTCTCTCTCCCTATCTTGCATTTTGTTTAGACGAGATTCTTACACTCTTCAACGATTGTTTTTGGCTGAACTCTGAATGTGAAAGTTAAGCTGCTTCTAAAGTAATTTGTATCGCTTGTTTTTGATGATCTCTTATTCATAGTTTAATTCCTAATTGTTGGAGTTTCATTCCCTCAATCGCATCGGGGGATACGACACATTTCTGGGACATGCCTTGTCGGTGACCTTTAGATATTAGCAACTACGAGGTTTACCCCAAAATCGGCTGAGAATGAAAGGCTTCTATAGCAAAGCACAAGTTGGTCGAGGCCTAAGGGAAGAAGACTAGAAAGGTGGTGTCATATGGTGAGTTAGTCTCTGCCAGTCTAGAGGTTCCTAAAGAAGAGGATTGGGATCTTCATTGTGTTCGTAAAGGGATCCACTATTTTAATCTCTTGATT |
| **AtPHS1** | AT5G54270 | AATGGGCAACCAGAAAAGAAATATGGATAAAAAAGAGATGGAATTAGAGATACTTCAAATCCAAAGCTGTTTCCTGATTGGCTAATCCCAACATCTCACTCTCTATCTATATCCCATCTCTGATTCTCCCACCTCTCTTCTCATCCACAAAATACTAACATAAAAGTCAAAGTCCCTGAGACCAATACTTTCACCAAACAGCAAAACAAGAAGAACAACAACACTAAGCAAAAAAGAAAAGCTCAAGCCGAGAGAGACAATGGCATCAACATTCACGAGCTCAAGCAGTGTTCTTACCCCAACAACATTCCTTGGCCAGACTAAAGCCTCAAGCTTTAACCCCCTTCGTGATGTTGTCTCTCTCGGATCTCCCAAGTACACTATGGGAAATGATCTTTGGTATGGACCTGACAGAGTGAAGTACTTAGGACCCTTTTCCGTTCAAACTCCGTCTTACCTCACCGGAGAATTCCCTGGCGATTATGGTTGGGACACCGCCGGTTTATCCGCAGACCCTGAAGCCTTTGCCAAAAACAGAGCTCTTGAGGTGATCCATGGGAGATGGGCAATGTTGGGAGCTTTTGGTTGCATAACCCCTGAAGTTCTTCAAAAGTGGGTCCGTGTGGACTTCAAAGAACCAGTCTGGTTCAAAGCCGGTTCACAAATCTTCTCCGAAGGCGGTTTGGACTACTTAGGCAACCCAAACCTAGTCCATGCTCAGAGCATTTTAGCCGTCCTTGGCTTCCAAGTCATCCTCATGGGTTTGGTTGAAGGTTTCCGCATCAACGGTCTTGATGGTGTTGGCGAAGGCAACGACTTGTACCCCGGTGGGCAATACTTTGACCCGTTGGGTCTCGCTGATGATCCAGTTACTTTTGCTGAGCTTAAGGTGAAAGAGATCAAGAACGGAAGATTGGCTATGTTCTCTATGTTTGGCTTCTTTGTTCAAGCCATTGTTACTGGAAAAGGTCCTTTGGAGAATCTCCTTGACCATCTTGACAACCCTGTTGCTAACAATGCGTGGGCTTTCGCAACTAAGTTTGCACCTGGAGCTTAAATTTTCAAGTCTTGATGTGATTCTTGTAATGATATGAACATAACCTTTCTTGTTCCTCTCAAATTACAAATCTTCTATTTGCTATGCACAGAAGAAGTCTCAAAAATACATGTCTCTCACAGCTAAATCTACAATGCTCTTAATCTAACATTTTGGGATCGTGATACTAATACAAAGCTAAGTGG |
| **AtPHS1** | AT4G25640 | ACCTTGACTCAGAGAGGCAGAGAGAGAGAGAGAGAGAGATATAGAAGAGAAGTGAGTGGCGTTTGGTTAATCATTTTCAGAAGATGGATCCGACGGCGCCGTTGCTTACACACGGTGGCGAAGTAGAGGAGGATTATGCTCCGGCGAGGAGCTGGACCGATGTGAAACGAGTCTTATCTACAGAGTCGGCCAAGTTGTGGATGATAGCTGCTCCTGTGGGGTTCAACATCATCTGCCAATACGGAGTTAGCTCAGTCACTAATATCTTTGTCGGTCACATCGGCGAAGTCGAGCTCTCCGCCGTCTCTATCTCTCTTTCCGTCATCGGTACCTTCTCCTTCGGTTTCTTGCTTGGTATGGGAAGTGCTCTTGAAACATTATGTGGTCAAGCATATGGAGCTGGTCAAGTCAATATGTTAGGAGTTTATATGCAGAGATCTTGGATTATCTTGTTCGTTTCTTGTTTCTTCCTCCTTCCTATTTACATCTTTGCCACGCCGGTTCTGAGACTACTCGGTCAAGCAGAGGAGATCGCTGTTCCAGCTGGACAGTTCACTCTTCTAACCATACCACAGCTCTTCTCACTGGCCTTTAATTTCCCAACCTCTAAGTTCCTTCAAGCACAGAGCAAAGTGGTTGCTATTGCTTGGATTGGATTTGTGGCTCTTTCCCTTCACGTTATTATGCTCTGGTTGTTTATAATCGAGTTTGGTTGGGGGACAAATGGTGCTGCTTTGGCGTTTAATATTACAAACTGGGGAACTGCAATTGCTCAAATTGTTTATGTGATTGGTTGGTGTAATGAAGGCTGGACTGGTTTATCTTGGTTGGCTTTTAAAGAGATTTGGGCTTTCGTTAGACTCTCCATTGCATCTGCTGTTATGCTTTGTCTTGAGATCTGGTATATGATGAGTATCATTGTTCTTACTGGCCGTCTTGACAATGCAGTTATCGCTGTTGATTCCCTTTCTATATGCATGAATATCAACGGCTTGGAGGCTATGTTATTCATCGGAATAAATGCAGCTATAAGTGTCCGTGTATCCAATGAGCTTGGCCTAGGCCGTCCACGAGCAGCTAAGTACTCTGTCTATGTCACGGTCTTCCAGTCTCTCCTCATTGGTCTTGTCTTTATGGTGGCTATCATCATAGCCAGAGACCATTTTGCTATCATCTTCACAAGCAGTAAAGTACTTCAGCGTGCAGTGTCTAAGCTAGCTTATCTTCTTGGTATAACCATGGTTCTCAACAGTGTGCAGCCGGTTGTTTCTGGTGTGGCTGTTGGAGGCGGTTGGCAAGGCTTAGTGGCCTATATCAACTTGGGCTGTTACTACATTTTTGGCCTTCCCTTTGGATATCTTCTTGGTTACATTGCAAACTTTGGAGTGATGGGACTTTGGTCTGGAATGATAGCCGGGACAGCGCTTCAAACGTTGTTACTGTTGATTGTTCTGTATAAGACAAACTGGAATAAAGAGGTGGAGGAGACGATGGAACGTATGAAGAAATGGGGAGGGAGTGAGACGACATCCAAGGATATACTTGCGTCAGGATGGCCGAGTGGTCTAAGGCGCCAGACTCAAGTTCTGGTCTTCGTAAGAGGGCGTGGGTTCAAACCCCACTTCTGACATTAGTTTTTTGTTACAGTTTCTGCGTGAGCTTTTTGTATTTACATTTCCTTGGCTTGTGTCCTAAAAATGCAACGTGTCTTCAAACTTTGTTTTGTTTGTGTGCAAATTCTTGCGGCGTTTTACATTTGC |
| **AtPHS1** | AT2G34420 | AATCACTCTCACCAGTCACAACCAAAAAAAAGAAAACACAAAAACAAAGATTATAATGGCTTCCTCAACCATGGCTTTGTCCTCCCCTGCCTTCGCCGGAAAGGCTGTGAAGCCTGCCGCATCAGATGTCCTCGGAAGCGGCCGTGTGACCATGAGGAAGACTGTCGCCAAGCCAAAGGGTCCATCAGGCAGCCCATGGTACGGATCTGACCGAGTCAAGTACTTGGGTCCATTCTCCGGCGAGCCCCCGAGCTACCTTACCGGTGAGTTCCCCGGTGACTACGGATGGGACACCGCTGGTCTATCCGCCGACCCAGAGACCTTCGCCAGGAACCGTGAGCTAGAAGTTATCCACAGCAGATGGGCCATGCTCGGAGCCCTAGGCTGCGTTTTCCCTGAGCTATTGGCTAGGAACGGAGTGAAGTTCGGAGAAGCGGTTTGGTTCAAGGCCGGTTCACAGATCTTCAGCGACGGAGGATTGGACTACTTGGGCAACCCGAGCTTGGTCCACGCTCAGAGCATCTTAGCCATTTGGGCTACTCAAGTTATCCTCATGGGAGCTGTTGAAGGCTACAGAGTCGCCGGAGATGGTCCATTGGGAGAAGCAGAGGACTTGCTTTACCCAGGTGGCAGCTTCGACCCATTGGGCCTCGCTACTGACCCCGAGGCTTTCGCAGAGTTGAAGGTGAAGGAGCTCAAGAACGGAAGGTTGGCTATGTTCTCTATGTTTGGATTCTTCGTTCAAGCCATCGTCACCGGAAAGGGACCTTTGGAGAACCTCGCCGACCATTTGGCCGACCCAGTCAACAACAACGCTTGGGCCTTCGCCACCAACTTCGTCCCCGGAAAGTGAGCGGCTGCTTATTATGTGAATGAGAGCAGAGAAAGAGAGTTTGTTTGTGGTCTATTCTATGTAAATTTGTGAACTTTCCTTTGTGGTATCTTTGTATAGAAATCGAACCTCTTTTTCTCTAACTGTGTTGGCGTTTTATCTGAAGACTCAATTGAATTGAAATATCCACTCTTGTAAAATTCATATGTTCTG |
| **AtPTST2** | AT3G16000.1 | AAAAAAAGTTTCAGTCTTTGATACCCAATTTCGTAGGATTTGTAGATATTTTCTTCAGTAATCGAAGGATATAACTCTGAGATTTGTGTAAGCATTCTTCTTTGAGATTTTCCGAGGTCACAAGTCAGTACACAAAGGAAGTTCCTTTTTTGGGTTTTGGGGTCTGCTTTGTGTCAGTGATTTTAGGATTGCTTCATTTGCCACACATTTCCAATCTCTTAAGTAGTTGGCGGCGAAGATGGGTTTCCTGATAGGGGGCTCTTGCTTCGTCCCTTCTGTTCCTCTACACTCTCGATTCCTTTCATCTCCTTCTTCTTCTTCTTCTTCTTCTCCTTCTTCGTCTCAGTTTGGGCTTCTGTGTTCGAGTAATGTCGCCAAGTTCAAGCGCCGACGACCAACATTGGCTTCTTTAAACCAGGAAGATGGGTACGAGTATGACGTTGCTTCCGCCAAAAGGAGGGCTTTCCTTCTTGTGGGTATCTCTGTTCTTCCCTTTTTGCAGCTTCGATCACCCGCTTTGGCTGATGAAAGAGGCAATGAGATAAAGACATCGAAGGTTGATCTAGAAACTGAGGTTGCAGTAGTTAGTGAAGGAACATCCCCAAATCCATTTCTGGCTCTCCTGAATGGTCTTGGAATTTTTAGTGCTGGCGTTCTTGGTGCACTTTATGCACTGGCTCGGCAAGATACAAAAGCTGCTGAGGAAACCATCGAATCTCTAAAGAACCAGTTGAAAGACAGAGAAAGAGCATTGGTTTTGAAGGAGAAAGATTTCGAGGCAAAACTGCAGCATGAGCAGGAAGAGCGGAAAAAGGAAGTAGAAAAGGCAAAAGAGGAACAGTTGTCATTGATCAACCAGTTGAATTCTGCAAAGGACTTGGTGACAGAATTAGGCCGGGAGCTAAGTAGCGAGAAGAAATTATGTGAGAAGCTTAAAGATCAAATCGAAAGTCTGGAAAATAGTCTGTCAAAGGCTGGCGAAGACAAAGAGGCACTAGAAACAAAGCTCAGAGAAAAGCTTGATTTGGTTGAAGGACTACAAGATCGGATCAACTTGCTTAGTTTGGAGCTGAAAGATAGTGAAGAAAAAGCTCAACGTTTTAATGCATCGTTGGCAAAAAAGGAAGCAGAATTGAAGGAACTCAACTCTATTTACACTCAAACTAGCCGAGATCTTGCTGAAGCGAAGTTAGAGATCAAACAGCAGAAGGAAGAACTCATAAGAACTCAGAGTGAATTGGACTCGAAGAACTCTGCGATTGAGGAGTTAAACACAAGAATAACAACTTTAGTGGCTGAGAAAGAGAGTTATATCCAGAAGCTTGATAGTATTTCAAAAGATTATAGTGCTTTGAAATTGACTTCTGAGACGCAAGCAGCTGCAGATGCAGAGCTCATCAGCAGGAAAGAGCAGGAGATTCAGCAGCTAAATGAAAATCTGGATCGTGCGCTAGATGATGTTAATAAAAGTAAAGACAAAGTTGCTGACTTAACTGAGAAGTACGAAGACTCGAAGAGAATGCTGGATATAGAACTGACTACAGTAAAAAATTTGAGACATGAACTTGAAGGAACAAAGAAAACACTGCAGGCATCTAGAGATCGGGTCTCTGACCTGGAAACGATGCTTGATGAGTCAAGAGCTTTGTGTTCAAAGTTAGAATCAGAGCTTGCTATAGTTCACGAAGAATGGAAGGAAGCTAAGGAAAGATATGAAAGGAACCTCGATGCTGAAAAACAAAAGAATGAGATTTCTGCTAGCGAACTTGCACTGGAGAAAGATCTTCGGAGGAGAGTTAAAGACGAGCTTGAGGGAGTAACTCATGAACTCAAAGAGTCTTCTGTCAAGAACCAGAGCCTCCAGAAGGAACTTGTGGAGATTTACAAGAAAGTTGAAACCAGTAACAAGGAATTGGAAGAGGAGAAAAAGACTGTTTTGTCGTTGAACAAAGAGGTGAAAGGAATGGAAAAGCAGATCTTGATGGAAAGGGAGGCGAGAAAATCCCTTGAAACAGATCTCGAAGAAGCTGTAAAGTCTTTAGATGAGATGAACAAGAACACATCAATACTGTCACGAGAGCTTGAGAAGGTGAATACCCATGCTTCAAACTTGGAGGACGAGAAAGAAGTACTTCAACGATCACTAGGAGAGGCAAAGAATGCATCAAAAGAAGCTAAGGAAAATGTGGAAGATGCACATATCCTCGTGATGAGTCTAGGAAAAGAAAGGGAAGTGCTAGAGAAGAAAGTGAAGAAGCTCGAGGAGGACTTGGGCTCTGCAAAGGGCGAGATACTGCGCATGAGGAGCCAACCGGATTCTGTAAAAGCTGTGAATAGTACAGACAACAAAGAGAAGAGCGACAACACGGTTACTGTGAAGAAAGTTGTCAGGAGGAGAAAGAGCAGTACCAGTTCTTGAAGAGAGAAGGTGATAATGACCAGAGCTTGCTTCTCTAATCATATAACGCAGATTATTTGTAGCCAATATTAAACCTTTGTGTAAATTTTGACAAGAGGCAAAATGGTTCTAGAGTTTCATCATTGAGATAATCCCAACAGTGATTATAGAAAGTATAAAATGAGAAGATAAATGCTTTGCATAGGCTATTTATATCCAAATTATGTATACATACCGAATTGTTAAAAGATGAAATTACATTTATTCTACAATTAAAACAGAAACTCCCTCAAGGAGTTTTCAG |
| **AtPTST2** | AT5G03420.1 | AATAAATGCTGGGAACATCGGAGCTTGAGAAAGTCCGGCCATGGCGACGATTTCTCAGATACCTTTCTCTATCTCCTTCCCTTGTTTCGAATTTCGGAAACCATCGTTCTACTATCATCAGCCTCAACTTTTTGTTTCGTACTTGAATTCCACGAAGAAGCATAGTTTTATCTGTTTCGCATGTTCCACCAAACAAACCAGAGTTCGTAAGAGAGTGAAGAGCAATGAGGAGCTTCGGAGTGAGATTATGCAGTTCGTTGCTTTGGCTGGGCTTCCTGAAGGTCACGTGCCGTCCATGAAGGAACTCTCCGCACATGGAAGAGTTGATCTTGCTAACATTGTAAGACGAAGAGGCTATAAATTCATCAAGGAACTTGTTGCAAACTCTGGTATGGAGGAAGACTGTAACGAGCTAGTCGCTGACTCTGAGGATAATAATACCAACATTGAAACAGGAGGAAGCCGAGCTTGTTTGGAAGATTCGTCAACAGATTTAAGTAAGGAGGCTGAAAAACAAGGAAGTTTGAGTAAAGATGAATCGTCACTGGCTGGAGTTTTGAGCTTGGAAAACTCTTTTTCTAACTTGGGTGACAGTAATCATTCAGGAGAAATCACAGAAAAAATTTTCAAGATTGAGAGTGTGGAGCTCAATGAGATAGCTGACATTGAAAACTCATCGTCTGAAGCTTCAGTATTTGCAAACCATTCTCAAGATTTATATGATACTTCAAGCTGTCCTGACATAGAAGCAGGCAATGTTTCGATGACAGAGGATGAGGAGGTCAACGATGTGGACAAAGATTTCTCTCTTACGTTTGATCATTACACCAGTCCTACCTCTAATCATTACACTAGTCCAGATCTCAATTCCATCAAACATGTTGACATTGCTACTGGATCCTCGTATGATCTGACCTCAGAGAATACCATGACAAATGTAGAGAATTTTCAGAATCAGCAGATTGATGATATTGCTGCAAATCGTTCTGGTAGTGCTGATGATAGTTTAGTTGAGTCTGAGGATAATGATTGGATGTCAGGGCTTTCTTCTTGCACTTCAAGCATAGAAGAAAAAACAACCAGATTTATTCAGAATGGATACTTGGATACAGTTGGTGCTGATGAAAATGACATACCCAATGAAAGCTGTCCTGAAGAAAGTTCAGAAACAACCAAAGGTGGTGAGTACATAGGAGACAGTCTTGGAGGTCCGAGAATTATGAGTACACCACTAAATGGAAGTGCCCTAGCATTGAAGGAGATAATCCATGCTACAGAAGTTAATAGCTCTGACAGGAATAGTGATCAGAGAGATGGGAGTGTTGGCTTGGACACGGATCCACATCATGAGACCAGGAAACGGGAAAATCAGGTTGAAATTGATCGGCTGAGGTTCATGCTGGATCAAAAAGAGCTGGAGTTGTCAAGGCTGAAAGAGCAGATTGAAAAAGAAAAGCTGTCTCTTTCAGTTCTCCAAAGACAGGCTGAAACAGAGATCCAAAAGGCCCAAATGCTTATCTCAGAGAAGGAAGTTGAATTGCAAGAAGCTGAGGAAAGTCTTTCAGGATTGCAAGAGGTTGTGATTGAATATTGCGGAGATGGTAACGCTGTGGAAGTGACTGGCAGTTTCAATGGGTGGCAACACCGAGTGGGAATGGAACTACAAGCATCCAAGTCAATAGGGAAACAGAAATGCTGGTCGACATTACTGTGGTTATATCCCGGTACATATGAGATAAAGTTCATCGTGGACGGGCAATGGATAACGGATCCTCAAAAAGATTCGGTTACAAGAGGACATATCTCAAACAACATTCTCAAAGTAGACAGCCAATGATCTCTTGCCTTTACTGATTTTTCGAGTCGAATTCACTCCAGATGATCGGTGCAGGCTTAAACAGGTGAAAAGAGCAGAGCAAAACATTTCAAAGGTTTATTAGCCTCTTCAATTTAAGTCTACGATGTTTTGCTTTTATTCTTTGAAGCTTAGGGATAGACTCTGTACAAAATTGTAATTTAAGGTAAAATAATACATTTATAAGGTTT |

**S-4:**

Table 7. Homologous protein.

| **Homologs of AT2G34420** | **Accession** | **Protein** | **Location** |
| --- | --- | --- | --- |
|  | AT1G29930 | LHCB1.3, AB140, CAB140, CAB1 | Chloroplast |
|  | AT1G29920 | AB165, CAB2, CHLOROPHYLL A/B-BINDING PROTEIN 2, LHCB1.1 | Apoplast,  Chloroplast,  Chloroplast  Thylakoid membrane, thylakoid |
|  | AT1G29910 | CAB3, LHCB1.2, AB180 | Chloroplast |
|  | AT2G34430 | DEG11, LHB1B1, LHCB1.4, | Chloroplast |
